# Brain-derived neurotrophic factor in VMH as the causal factor for and therapeutic tool to treat visceral adiposity and hyperleptinemia in type 2 diabetic Goto–Kakizaki rats

**DOI:** 10.3389/fnsyn.2013.00007

**Published:** 2013-10-02

**Authors:** Fumihiko Maekawa, Ken Fujiwara, Masako Toriya, Yuko Maejima, Takashi Nishio, Yukiyasu Toyoda, Keiko Nohara, Takashi Yashiro, Toshihiko Yada

**Affiliations:** ^1^Division of Integrative Physiology, Department of Physiology, Jichi Medical UniversityShimotsuke, Japan; ^2^Molecular Toxicology Section, Center for Environmental Health Sciences, National Institute for Environmental StudiesTsukuba, Japan; ^3^Division of Histology and Cell Biology, Department of Anatomy, Jichi Medical UniversityShimotsuke, Japan; ^4^Laboratory of Neuropsychopharmacology, Graduate School of Environmental and Human Sciences, Meijo UniversityNagoya, Japan; ^5^Department of Pathobiochemistry, Faculty of Pharmacy, Meijo UniversityNagoya, Japan

**Keywords:** BDNF, type 2 diabetes, adiposity, visceral fat, hyperleptinemia, glucose, VMH

## Abstract

We previously reported that the type 2 diabetic Goto–Kakizaki (GK) rats at young adult ages (6–12 weeks) exhibited increased visceral fat mass and hyperleptinemia, due to hyperphagia caused primarily by neuropeptide Y (NPY) overexpression in the hypothalamic arcuate nucleus. Later, we found that GK rats continued to exhibit mesenteric fat accumulation and hyperleptinemia at least until 26 weeks of age, while hyperphagia and NPY overexpression ceased at 15 weeks of age. Therefore, we hypothesized that the long-lasting fat accumulation and hyperleptinemia are due to unidentified brain dysfunction other than NPY overexpression. In GK rats aged 26 weeks, glucose transporter-2 (GLUT2) mRNA expression in ventromedial hypothalamus (VMH) was markedly reduced in parallel with significant decreases in brain-derived neurotrophic factor (BDNF) mRNA level and BDNF-expressing cell numbers in the VMH. Pharmacologic inhibition of glucose utilization reduced BDNF mRNA expression in VMH *in vivo* and *in vitro*. The results suggested that impaired glucose utilization caused the reduction of BDNF. On the other hand, intracerebroventricular injection of BDNF for 6 days ameliorated hyperleptinemia in a long-lasting manner concurrently with feeding suppression in GK rats. Restricted feeding paired to BDNF-treated rats reduced plasma leptin level only transiently. BDNF treatment also reduced mesenteric fat mass in GK rats. These results reveal a novel action mode of BDNF to long-lastingly counteract visceral adiposity and hyperleptinemia in addition to and independently of its anorexigenic action. These results suggest that visceral fat accumulation and hyperleptinemia are at least partly due to the reduction of BDNF in VMH primarily caused by impaired glucose utilization in GK rats. The BDNF supplementation could provide an effective treatment of visceral obesity, hyperleptinemia and leptin resistance in type 2 diabetes.

## Introduction

The Goto–Kakizaki (GK) rat was established as an animal model for type 2 diabetes by selecting Wistar rats which exhibited glucose intolerance (Goto et al., 1976) and has been widely used to investigate the mechanisms of glucose intolerance and complications of hyperglycemia (Yagihashi et al., 1982; Picarel-Blanchot et al., 1996; Moreira et al., 2007). It has been reported that glucose intolerance in GK rats is mainly caused by reduced β-cell mass and impaired glucose-induced insulin secretion in β-cell (Portha et al., 1991). Glucose intolerance of GK rats is also thought to be partly due to impaired insulin sensitivity (Farese et al., 1994).

Our particular concern in this study is how impaired insulin sensitivity develops and is sustained in GK rats. We previously reported that impaired insulin sensitivity and increased visceral fat mass occurred in parallel in young adult (6–12 weeks) GK rats (Maekawa et al., 2006a). The result indicated a possibility that the increased visceral fat mass induced impairment of insulin sensitivity. In young adult (6–12 weeks), the increased fat mass was initiated by hyperphagia which was caused by enhanced neuropeptide Y (NPY) mRNA expression and impaired intracellular signaling of leptin in the hypothalamic arcuate nucleus (ARC) (Maekawa et al., 2006a). In GK rats, it has been also reported that insulin sensitivity is impaired not only at young adults but at middle-aged adults (Ndisang and Jadhav, 2009). Middle-aged GK rat in our colony (22–35 weeks) exhibited hyperleptinemia and higher mesenteric fat weight, while hyperphagia and overexpression of NPY mRNA in ARC were no longer observed (Maekawa et al., 2006a). Therefore, it is speculated that the long-lasting fat accumulation and hyperleptinemia at middle-age are due to decreased energy expenditure.

In this study, we focused on the relationship of brain-derived neurotrophic factor (BDNF) to fat accumulation and plasma leptin level in GK rat at middle-aged adult. In addition to well-defined role of BDNF in the regulation of development, survival, differentiation and synaptic plasticity in the nervous system (Tapia-Arancibia et al., 2004), it is also implicated in feeding behavior and energy balance (Noble et al., 2011; Rios, 2013). Heterozygous BDNF mutant mice and brain-specific BDNF knockout mice, in which the BDNF gene is selectively deleted in the brain after birth, showed increased food intake and body weight (Lyons et al., 1999; Kernie et al., 2000; Rios et al., 2001). Intracerebroventricular (icv) BDNF injection markedly reduced body weight in several obesity models by reducing appetite (Pelleymounter et al., 1995) and increasing energy expenditure (Nakagawa et al., 2000). In the hypothalamus, BDNF-expressing neurons are mainly localized in ventromedial hypothalamic nucleus (VMH) and paraventricular nucleus (PVN) (Noble et al., 2011). Since BDNF knockout in medial basal hypothalamus containing VMH induced hyperphagia and obesity, the BDNF neurons in this region is thought to be critical for regulating energy metabolism (Unger et al., 2007). BDNF neurons of VMH possibly project to the neurons expressing corticotrophin-releasing hormone (CRH) in PVN, since the CRH neurons express TrkB, a receptor for BDNF, and icv injection of BDNF increases CRH mRNA (Toriya et al., 2010). BDNF injection decreased respiratory quotient and increased rectal temperature, and these effects were antagonized by simultaneous treatment with α-helical CRH_9–41_, a CRH receptor antagonist (Toriya et al., 2010). These results suggested that the projection of BDNF neurons to CRH neurons in PVN plays a critical role in energy expenditure.

It has been reported that the BDNF expression in hypothalamus is regulated by several factors, which include neurotransmitters such as melanocortins (Nicholson et al., 2007; Vanevski and Xu, 2013), metabolic factors such as leptin (Komori et al., 2006), insulin and glucose (Unger et al., 2007), and environmental cues such as stress and environmental enrichment (Cao et al., 2010). In addition, BDNF level in hypothalamus is altered in obesity and/or diabetes models such as db/db, agouti yellow (Xu et al., 2003) and SF-1 knockout mice (Tran et al., 2003). These reports suggest that metabolic changes in obesity and diabetes result in and/or result from the reduction of BDNF expression.

Here, we found that BDNF expression is reduced specifically in VMH in GK rats at middle-age. We investigated the mechanism underlying the reduction of BDNF in VMH and examined whether BDNF supplementation induces lipolysis and ameliorates visceral obesity.

## Materials and methods

### Animals

The GK rats purchased from Japan SLC (Shizuoka, Japan) were maintained by breeding in the Center for Experimental Medicine, Jichi Medical University. Adult male or pregnant Wistar rats were purchased from Japan SLC. The rats were housed under a controlled temperature (26°C) and photoperiod (12L:12D). The rats received pellet-type food (CE-2, Japan CLEA, Tokyo, Japan) and tap water *ad-libitum*. The animal protocols were approved by the Jichi Medical School Institute of Animal Care and Use Committee and were in accord with the Japanese Physiological Society's guidelines for animal care.

### mRNA extraction from brain tissues and cDNA synthesis

A portion of hypothalamus in each rat was dissected using a brain slicer. The coronal slice of 2-mm thickness covering the anterior part of VMH to the posterior part of ARC was obtained. The tissues of the VMH and ARC were dissected with incisions, and homogenized with TRIzol (Invitrogen, Carlsbad, CA). Total RNAs were extracted following the protocol indicated by the manufacturer. DNase (1 U/10 μ l RNA solution, Promega, Madison, WI) was added and the mixtures were incubated for 1 h at 37°C. Following the inactivation of DNase by heat, cDNA was synthesized from 2 μ g total RNA with SuperscriptII Reverse Transcription Kit (Invitrogen) utilizing oligo(dT)_20_ primer.

### Mixed culture of cells from the mediobasal hypothalamus and mRNA extraction

The mediobasal hypothalamic tissue was isolated from the brain of 6-day-old pups of Wistar rats, followed by dissociation of neurons according to the procedures reported previously (Kohno et al., 2007) with slight modification. Briefly, brain slices in 1 mm-thickness were prepared, from which the tissues containing the VMH and ARC were obtained. The 5–6 dissected tissues were mixed and washed with 10 mM HEPES-buffered Krebs-Ringer bicarbonate buffer (HKRB) [(in mM): NaCl 129, NaHCO_3_ 5.0, KCl 4.7, KH_2_PO_4_ 1.2, CaCl_2_ 2.0, MgSO_4_ 1.2 and HEPES 10 at pH 7.4] containing 10 mM glucose. Then they were incubated in HKRB supplemented with 20 units/ml papain (Sigma-Aldrich, St. Louis, MO), 0.015 mg/ml DNase, and 0.75 mg/ml BSA for 12 min at 36°C in a shaking water bath, followed by graded trituration. The cell suspension was incubated on ice for 5 min and a supernatant was centrifuged at 500 rpm for 5 min. The pellet was resuspended in the culture medium containing 50% minimal essential medium, 25% Hank's balanced salt solution, 25% horse serum (#12360-038, #24020-117, #16050-122, Gibco BRL) and 10 mM glucose. The cells were distributed onto 96-well plates and short-term cultured. Each well-contained ~20% of cells removed from the brain of a pup. Three h after the treatment with 2-deoxy-d-glucose (2-DG) at various dosages, total RNA was extracted and cDNA was synthesized.

### mRNA measurements by fluorescence real-time RT-PCR

Quantification of mRNAs was carried out using ABI PRISM ®7900-HT system (Applied Biosystems, Foster City, CA). The glyceraldehyde-3-phosphate dehydrogenase (GAPDH) level was measured as an internal control. PCR primer sets for amplification and TaqMan®probes with carboxyfluorescein in 5′end and carboxytetramethyl-rhodamine in 3′end were purchased from Sigma Genosys (Hokkaido, Japan) (Table A1). To detect GLUT8, SybrGreen PCR was performed. Dividing each mRNA level by the GAPDH level resulted in a normalized mRNA value. The fluorescence real-time PCR was performed by the method as described previously (Maekawa et al., 2006a). Dividing each mRNA level by the GAPDH level resulted in a normalized mRNA value.

### Icv cannulae implantation

Rats were anesthetized with Avertin (a mixture of 2,2,2-tribromoethanol (T4840-2, Sigma-Aldrich) and Tert-amyl alcohol (24,048-6, Sigma-Aldrich), 200 mg/kg, intraperitoneal). In a stereotaxic frame, a 23-gauged stainless steel guide cannula was inserted into the brain with the tip in the third cerebral ventricle, and secured to the skull with screws and cement. The cannula tip was located at 0.9 mm caudal to the bregma and 7.0 mm below the skull. After surgery, rats were allowed to recuperate for 7 days. Handling of the operated animals was performed for 10 min everyday. For injecting substances, the internal cannula was inserted into the guide cannula and injection was executed for 1 min under free-moving conditions.

### Immunohistochemistry

A chicken anti-human BDNF polyclonal antibody (1:100, G1641, Promega), a sheep anti-α-MSH antibody (1:30,000, AB5087, Chemicon, Temecula, CA), a biotinylated rabbit anti-chicken IgY (1:300, G2891, Promega), a biotinylated rabbit anti-sheep IgG (1:300, BA-6000, Vector Laboratories, Burlingame, CA) were used. Rats were intracerebroventricularly treated with 10 μ l colchicine (100 μg/10 μ l in distilled water). Forty-eight h after injection, rats were perfused transcardially with 100 ml of 2% paraformaldehyde in 50 mM phosphate buffer (PB), pH 7.5, followed by 50 ml of 50 mM phosphate-buffered saline under deep urethane anesthesia. Brains were removed, postfixed by the same fixative for 2 h, cryoprotected with 30% sucrose in 50 mM PB for 2–3 days at 4°C, sectioned coronally at 40-μ m thickness with a freezing microtome. The sections collected at every 160-μ m interval were used. The localization of the target proteins was determined as described previously (Maekawa et al., 2006b).

### Icv BDNF injection, plasma hormone and metabolites levels

Wistar and GK rats were intracerebroventricularly injected with BDNF (15 μ g/5 μ l saline/head) or vehicle once a day for 6 days. Plasma samples were obtained at the day before injection, the 6th, 10th, 21st day after injection. Blood was withdrawn from tail vein under unanesthetized condition. Glucose level was determined by a conventional blood glucose-measuring device (Glucocard, Arkray, Kyoto, Japan). Intraperitoneal glucose tolerance test (IpGTT) was performed under overnight fasting conditions. Glucose (1 g/kg body weight) was injected and then blood samplings from the tail vein were performed up to 3 h. For hormonal assay, plasma was immediately separated. Plasma leptin and insulin concentrations were measured using ELISA kits for leptin and insulin, respectively (#200728 and #200718, Morinaga Institute of Biological Science, Yokohama, Japan). Plasma non-esterified free fatty acid (NEFA) concentration was measured using an assay kit for NEFA (#279-75401 NEFA *C*-test Wako, Osaka, Japan).

### Measurements of food intake and body weight combined with pair-feeding

Food intake over 24 h was calculated by weighing the remaining food pellets and body weight was measured between 11:00 and 12:00 h every day. For pair-feeding experiment, the amount of food consumed by the BDNF-treated group over the course of 24 h was measured at 11:00 h, and a corresponding amount of pellets was given to the pair-fed group over a 24-h period.

### Measurement of fat masses

At the completion of each experiment, interscapular, epididymal, mesenteric, and perirenal fat were dissected and weighed. Fat masses were calculated as percentage of body weight.

### Data analyses

**Figure 1:**
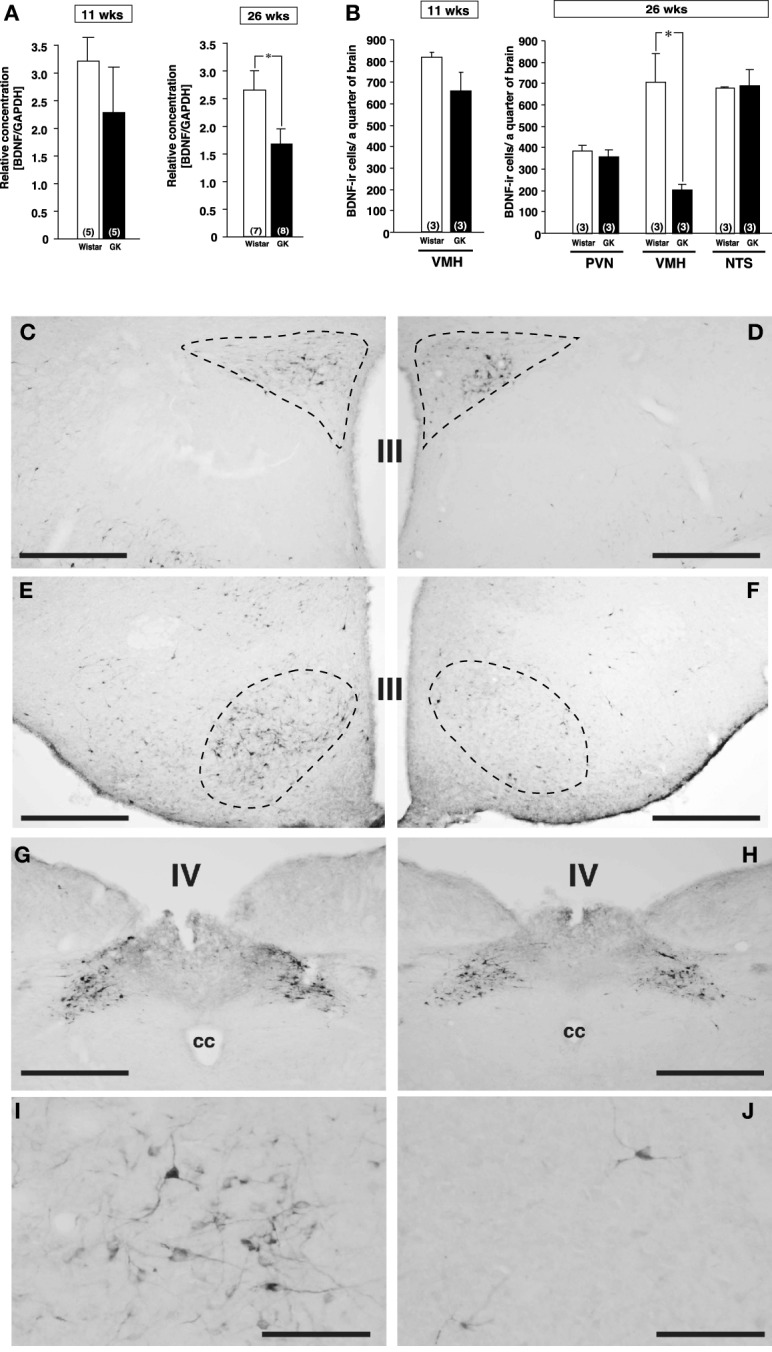
**Expressions of BDNF mRNA and BDNF-immunoreactive cells in Wistar and GK rats at 11 and 26 weeks of age. (A)** BDNF mRNA levels in VMH of Wistar and GK rats. Significant reduction of BDNF mRNA level was found in GK rats at 26 weeks of age (^*^p < 0.05, Student's *t*-test). **(B)** At 26 weeks, significant reduction of the number of BDNF-immunoreactive neurons was found in VMH, but not PVN and NTS, of GK rats (^*^p < 0.05, Two-Way ANOVA with Tukey's *post-hoc* test). This reduction in VMH was not different at 11 weeks. **(C–J)** Representative images of BDNF-immunoreactive neurons in Wistar rats **(C,E,G,I)** and GK rats **(D,F,H,J)** at 26 weeks of age. Broken lines in each image indicate the outline of PVN **(C,D)** and VMH **(E,F)**. No apparent difference was found in the PVN **(C,D)** and NTS **(G,H)**. By contrast, the number of BDNF-immunoreactive neurons in the VMH of GK rats **(F)** was fewer than that of Wistar rats **(E)**. At higher magnification, the difference between Wistar **(I)** and GK **(J)** rats was obvious. III; the third ventricle, IV; the fourth ventricle, cc; central canal. Scale bar = 500 μm **(C–H)** or 100 μm **(I** and **J)**.

All data are expressed as mean ± SEM. The number of animals used is indicated in the parenthesis. One-Way ANOVA with Holm's *post-hoc* test was used for Figures 3D, 4, 5A,B, 6, 7A,B, Table 2. Two-Way ANOVA with Tukey's *post-hoc* test was used for Figures 1B, 8, and Table 1. Other data was analyzed by Student's *t*-test with Microsoft Excel 2008 for Mac (Microsoft, Redmond, WA). All statistical analyses except Student's *t*-test were performed using R (The R Foundation for Statistical Computing, Vienna, Austria). *p* < 0.05 was considered significant.

**Table 1:** **Interscapular, epididymal, mesenteric and perirenal fat weights (% body weight) in control Wistar and diabetic GK rats at 11 and 26 weeks of age**.

**Age**	**Strain**	**No.**	**Weight of fat pad (%BW)**			
			**Interscapular**	**Epididymal**	**Mesenteric**	**Perirenal**
11 weeks	Wistar	6	0.09 ± 0.01	1.47 ± 0.08	0.63 ± 0.05	0.93 ± 0.04
	GK	5	0.21 ± 0.02^*^	1.04 ± 0.05^*^	1.08 ± 0.04^*^	1.78 ± 0.11^*^
26 weeks	Wistar	5	0.08 ± 0.02	1.52 ± 0.11	0.89 ± 0.05	1.55 ± 0.14
	GK	6	0.12 ± 0.01	1.16 ± 0.07^*^	1.27 ± 0.12^*^	1.97 ± 0.11^*^

* p< 0.05 vs Control Wistar rats (Two-Way ANOVA with Tukey's post-hoc test).

## Results

### Metabolic indices of GK rats at 26 weeks of age

**Figure A1:**
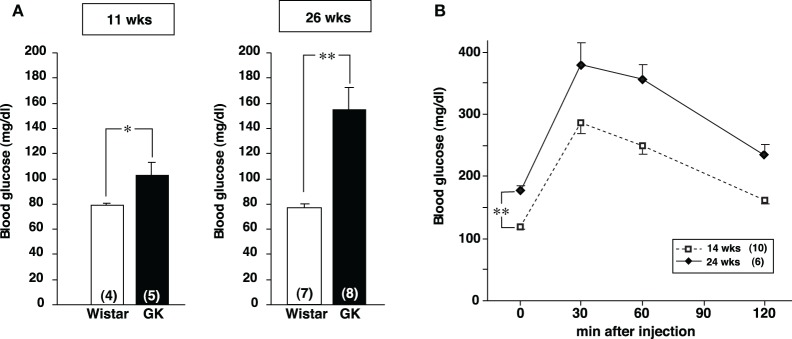
**Glycemic profiles of GK rats. (A)** The blood glucose level under fed conditions in Wistar and GK rats at the age of 11 and 26 weeks. The blood glucose level in GK rats was significantly higher than that in Wistar rats at both 11 and 26 weeks (^*^p < 0.05 and ^**^p < 0.01, respectively, Student's *t*-test). The difference was greater at 26 weeks. **(B)** Profiles of IpGTT in GK rats at 14 and 24 weeks. One g/kg body weight glucose (0.1 g/ml in distilled water) was injected at 0 min and blood glucose was measured at 0, 30, 60, and 120 min after injection. The glucose intolerance was progressed in GK rats at 24 weeks of age (^**^p < 0.01, Student's *t*-test).

Casual blood glucose level in GK rat was significantly higher than that in Wistar rat at 11 weeks and it increased further at 26 weeks (Figure A1A). Intraperitoneal glucose tolerance test in GK rats revealed that glucose intolerance progressed at 24 weeks, compared to 14 weeks of age (Figure A1B). Mesenteric and perirenal fat weights were larger in GK rats than in Wistar rats at 11 and 26 weeks of age (Table 1).

### Reduced BDNF expression in VMH of GK rats at 26 weeks of age

By comparison between Wistar and GK rats at 11 and 26 weeks of age, BDNF mRNA level examined by real-time RT-PCR was found to be reduced in VMH of GK rat specifically at 26 weeks of age (Figure 1A, *p* < 0.05). Furthermore, immunohistochemistry revealed marked reduction in the number of BDNF expressing neurons selectively in VMH but not in PVN and nucleus tractus solitarius (NTS) of GK rat at 26 weeks of age (Figures 1B–J, *p* < 0.05 in **B**).

### Mechanism underlying the reduced BDNF expression in VMH of GK rats

**Figure 2:**
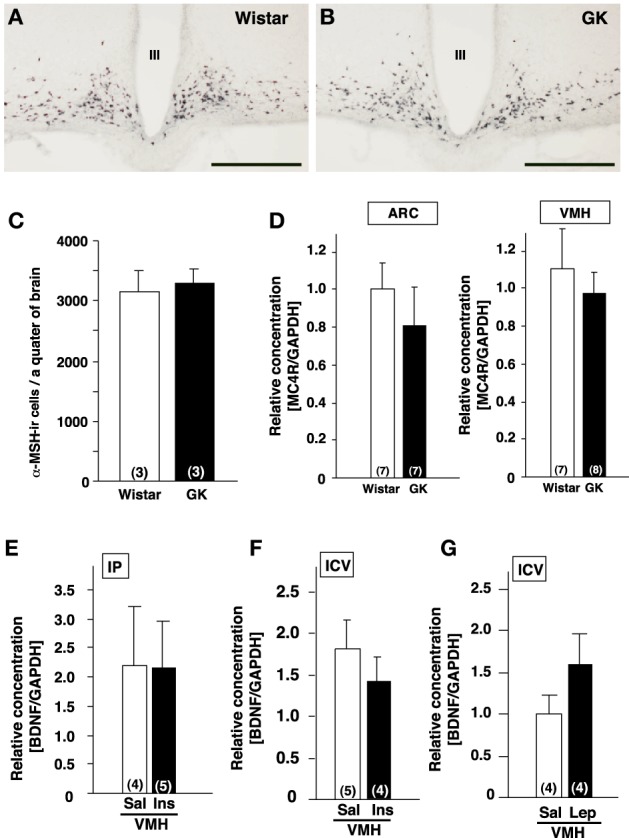
**Lack of alterations of melanocortines and effects of insulin and leptin on BDNF mRNA expression in GK rats. (A,B)** Representative images of α-MSH-immunoreactive neurons in ARC of Wistar **(A)** and GK **(B)** rats. III; the third ventricle, Scale bar = 500 μm. **(C)** Numbers of α-MSH-immunoreactive neurons in Wistar and GK rats. **(D)** MC4R mRNA expressions in ARC and VMH were not different between Wistar and GK rats. **(E)** Repeated intraperitoneal injection of insulin (1 U/kg body weight, twice a day for 3 days) in GK rats did not alter BDNF mRNA level in VMH. **(F)** Single icv injection of insulin (10 mU) in Wistar rats did not alter BDNF mRNA levels in VMH. **(G)** Repeated icv injection of leptin (5 μ g, twice a day for 3 days) to the fasting Wistar rats had no significant effect on BDNF mRNA levels in VMH of Wistar rats.

α-MSH, melanocortin-4 receptor (MC4R), insulin, leptin and glucose have been reported to affect BDNF expression. Hence, we examined possible involvement of these factors in reduction of BDNF expression in GK rat. Based on the previous report showing a link between BDNF and α-MSH (Xu et al., 2003; Nicholson et al., 2007), we examined a possible alteration of melanocortin system in GK rats. By immunohistochemistry, localization of α-MSH-immunoreactive neurons in the ARC of GK rats was identical to that in control Wistar rats (Figures 2A,B). The cell numbers of α-MSH-immunoreactive neurons in the ARC were not different between GK and Wistar rats (Figure 2C). Similarly, no difference in MC4R mRNA expression was found in the ARC and VMH in GK rats (Figure 2D).

Next, the possible association of insulin and/or leptin with BDNF reduction was examined. We tested whether the supplementation of insulin would rescue BDNF reduction in GK rats. GK rats were treated with intraperitoneal administration of insulin (1 U/kg body weight) twice a day for 3 days, which failed to restore the BDNF mRNA levels in the VMH (Figure 2E). To examine the acute effect of insulin to increase BDNF mRNA level in the VMH, single icv injection of insulin (10 mU/10 μ l saline) to Wistar rats was performed. However, it did not increase the BDNF mRNA expression (Figure 2F). Icv administration of leptin (5 μ g, twice a day for 3 days) in Wistar rats under fasting condition failed to alter BDNF mRNA level (Figure 2G).

**Figure 3:**
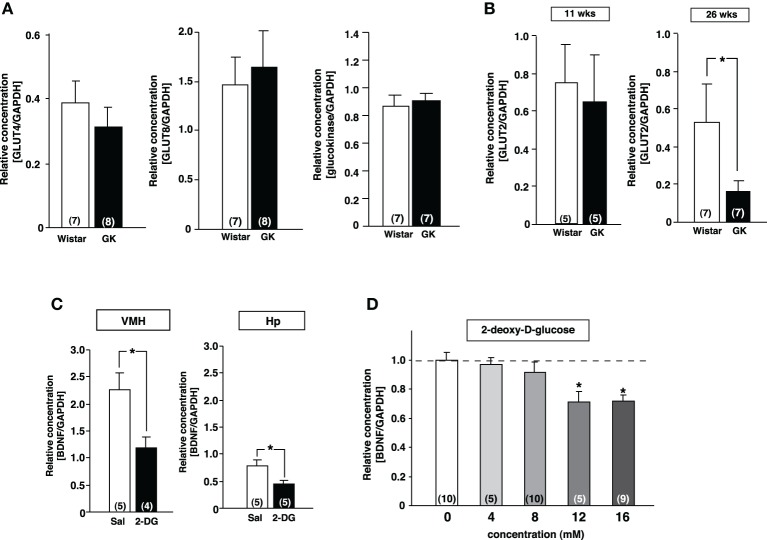
**Reduction of GLUT2 paralleled with reduction of BDNF expression in GK rats. (A)** GLUT4, GLUT8 and gluokinase mRNA expressions in VMH were unchanged in GK rats at 26 weeks. **(B)** GLUT2 mRNA expression in VMH was significantly reduced in GK rats at 26 weeks, but not at 11 weeks (^*^p < 0.05, Student's *t*-test). **(C)** Effects of icv 2-DG injection on BDNF mRNA levels in VMH were determined at 2 h after icv injection of 2-DG (5 mg/10 μ l) or saline (Sal). BDNF mRNA expression in VMH and hippocampus (Hp) was significantly reduced by 2-DG (^*^p < 0.05, Student's *t*-test). **(D)** Treatment with 2-DG for 3 h dose-dependently suppressed BDNF mRNA level in cultured mediobasal hypothalamic cells. The effects of 12 and 16 mM 2-DG were significant (^*^p < 0.05, One-Way ANOVA with Holm's *post-hoc* test).

Since transcription of BDNF in the VMH has been reported to be regulated by glucose (Unger et al., 2007), we examined the mRNA expression of genes related to the glucose utilization in the VMH of GK rats using real-time RT-PCR. There was no difference in GLUT4, GLUT8 and glucokinase mRNA expressions between Wistar and GK rats (Figure 3A). In contrast, the GLUT2 mRNA level was significantly reduced at 26 weeks compared to 11 weeks of age (Figure 3B). To test the possibility that reduction of glucose utilization affects BDNF mRNA level in the VMH, 2-DG (5 mg/10 μ l saline), an inhibitor of glycolysis, was administered icv to Wistar rats. 2-DG markedly reduced BDNF mRNA level in the VMH as well as in hippocampus at 2 h after treatment (Figure 3C). In a parallel *in vitro* experiment, bath application of 2-DG to primary cultured neurons of medial basal hypothalamus including the VMH dose-dependently reduced BDNF mRNA expression (Figure 3D). These results indicate that lowered glucose utilization in the VMH directly reduces BDNF mRNA expression.

### Effect of BDNF treatment on plasma leptin level and fat mass in GK rats

**Figure 4:**
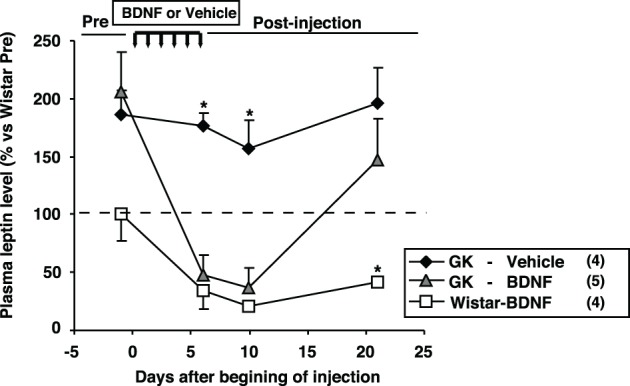
**Effect of icv BDNF treatment daily for 6 days on plasma leptin levels in GK rats.** Plasma leptin levels at 0, 6th, 10th, 21st days after beginning of BDNF or vehicle injections. BDNF treatment significantly reduced plasma leptin level in GK rats. ^*^p < 0.05 vs. other groups (One-Way ANOVA with Holm's *post-hoc* test).

The effect of daily treatment with BDNF (15 μ g/5 μ l saline/head) for 6 days on plasma leptin level and fat mass was examined in Wistar and GK rats at 26 weeks. Plasma leptin level in GK rats (GK-Vehicle group and GK-BDNF group) was significantly higher than that in Wistar rat before treatment (Day 0) (Figure 4), confirming our previous report (Maekawa et al., 2006a). GK rats subjected to treatment with saline revealed higher plasma leptin level during experiment (GK-Vehicle group, Figure 4). By contrast, the treatments with BDNF significantly decreased plasma leptin level in both Wistar and GK rats (Wistar-BDNF and GK-BDNF groups, respectively) at the timing of termination of treatment (Day 6) and at 4 days after termination of treatment (Day 10) (*p* < 0.01, Figure 4). Plasma leptin level at 15 days after termination of treatment (Day 21) returned to the level before treatment in GK rats of GK-BDNF group, while it was still low in Wistar-BDNF group. At Day 21, weights of fat mass in all groups were measured (Table 2). The weight of mesenteric fat in BDNF-treated GK rats (GK-BDNF group) was significantly lower than that in saline-treated GK rats (GK-Vehicle group, *p* < 0.05), although it was higher than that in BDNF-treated Wistar rats.

**Table 2:** **Effect of BDNF on interscapular, epidiymal, mesenteric and perirenal fat weights (% body weight) in GK rats**.

**Strain**	**Treatment**	**No.**	**Weight of Fat pad (%BW)**			
			**Interscapular**	**Epididymal**	**Mesenteric**	**Perirenal**
GK	Vehicle	6	0.15 ± 0.01	1.02 ± 0.11	1.14 ± 0.08	1.18 ± 0.14
GK	BDNF	5	0.17 ± 0.02	0.80 ± 0.07	0.80 ± 0.09^*^	0.97 ± 0.17
Wistar	BDNF	4	0.05 ± 0.01^*^	0.99 ± 0.08	0.55 ± 0.06^*^	0.56 ± 0.07^*^

Fat weights of saline-treated GK rats (GK-Vehicle group, 5 μ l saline/head, n = 6), BDNF-treated GK rats (GK-BDNF group, 15 μ g/5 μ l saline/head, n = 5) and BDNF-treated Wistar rats (Wistar-BDNF group, 15 μ g/5 μ l saline/head, n = 4) at Day 21 after the beginning of injections were examined. Interscapular:

* p < 0.05 vs. other groups, Mesenteric:

* p < 0.05 vs. GK-Vehicle, Perirenal:

* p < 0.05 vs. GK-Vehicle, One-Way ANOVA with Holm's post-hoc test.

### Effect of BDNF treatment on food consumption, body weight, glucose tolerance, and NEFA level in GK rats

**Figure 5:**
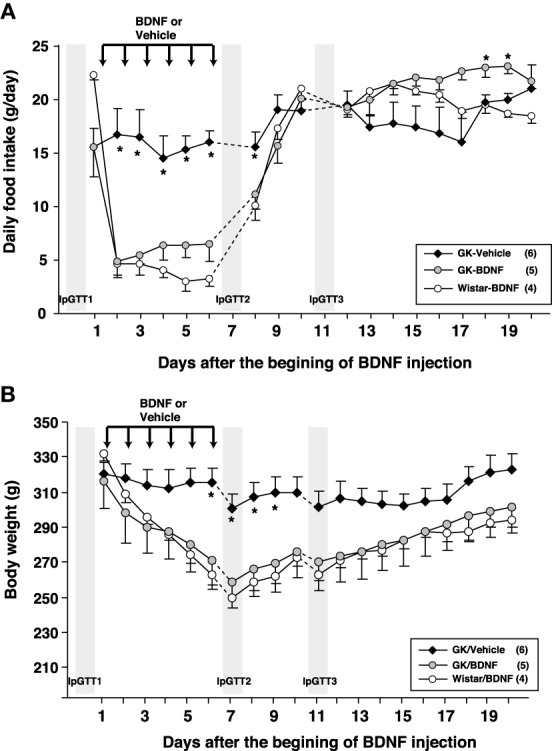
**Effect of BDNF treatment on food intake and body weight in GK rats. (A)** Daily food intake was examined before and after BDNF treatment (15 μ g/5 μ l saline/head) for 6 days. BDNF treatment significantly reduced daily food intake during period of treatment in both GK-BDNF and Wistar-BDNF groups. ^*^p < 0.05 vs. other groups (One-Way ANOVA with Holm's *post-hoc* test). **(B)** Body weight was examined before and after treatment of BDNF for 6 days. BDNF treatment significantly reduced body weight in both GK-BDNF and Wistar-BDNF groups. ^*^p < 0.05 vs. other groups (One-Way ANOVA with Holm's *post-hoc* test).

**Figure 6:**
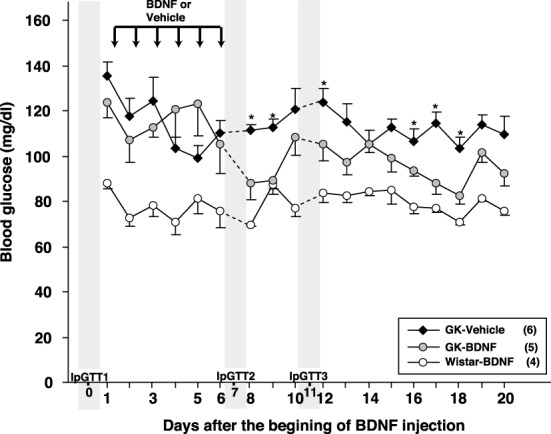
**Effect of BDNF treatment on casual blood glucose level in GK rats.** Although BDNF did not alter blood glucose level during treatment, significant reduction of blood glucose level was observed after termination of BDNF treatment in GK-BDNF group (^*^p < 0.05 vs. other groups, One-Way ANOVA with Holm's *post-hoc* test).

**Figure 7:**
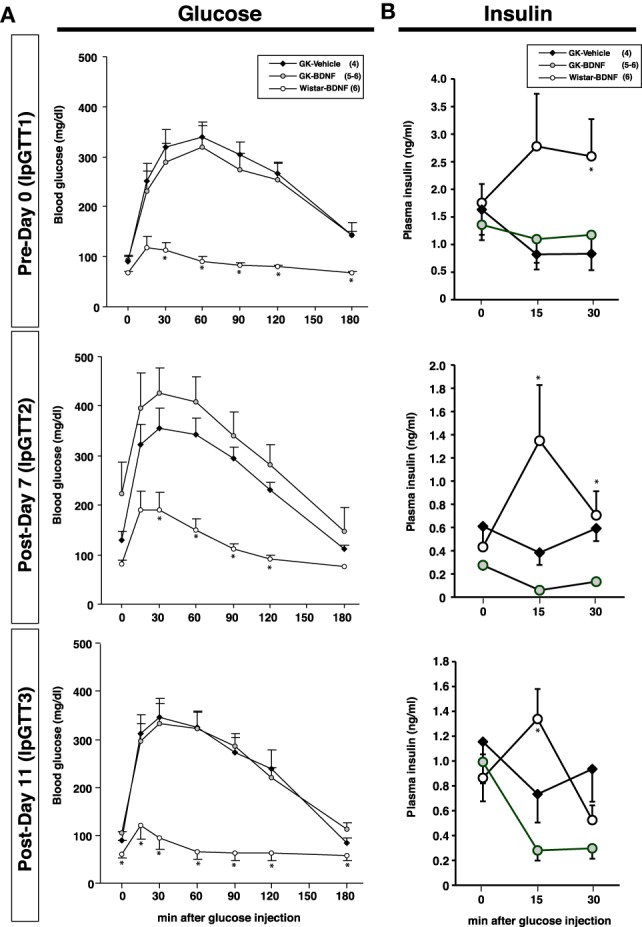
**Intraperitoneal glucose tolerance test before and after BDNF treatment in GK rats.** BDNF treatment did not attenuate glucose intolerance **(A)** and impaired insulin release **(B)** during glucose tolerance test in GK rats throughout experiment. **(A)** ^*^p < 0.05 vs. GK-Vehicle and GK-BDNF (One-Way ANOVA with Holm's *post-hoc* test). **(B)** ^*^p < 0.05 vs. GK-Vehicle in Day 0 (30 min), Day 7 (15 min), ^*^p < 0.05 vs. GK-BDNF in Day 7 (15 min, 30 min) and 11 (15 min), One-Way ANOVA with Holm's *post-hoc* test.

**Figure 8:**
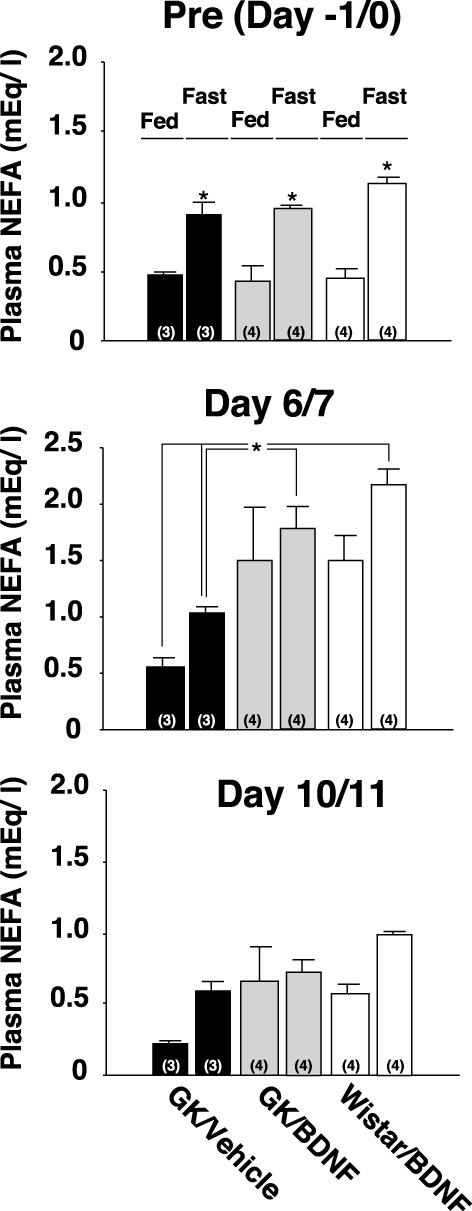
**Effect of BDNF on NEFA levels before and after treatment to GK rats in fed and fasted conditions.** Plasma NEFA levels in fed and 12 h-fasted conditions at time points before treatment (Day –1/0), immediately after termination of treatment (Day 6/7), and 4/5 days after termination of treatment (Day 10/11) were measured. Plasma NEFA level was higher in fasted condition at all time points measured in all groups [Pre(Day –1/0), *p* < 0.05 vs. fed conditions, Two-Way ANOVA with Tukey's *post-hoc* test]. At time point immediately after termination of treatment (Day 6/7), plasma NEFA levels in Wistar-BDNF and GK-BDNF groups were increased both at fed and fasting conditions. Especially, plasma NEFA levels in Wistar-BDNF and GK-BDNF groups at fasting condition were significantly higher than those in GK-Vehicle group at fasting condition (^*^p < 0.05, Two-Way ANOVA with Tukey's *post-hoc* test). Such tendency continued to Day 10/11 but there was no significant difference at this time point.

Daily food intake was significantly lowered both in Wistar-BDNF and GK-BDNF groups during BDNF treatment (Figure 5A). After termination of treatment, the food intake in Wistar-BDNF and GK-BDNF groups rebounded to the same level as that in GK-Vehicle group (Figure 5A). BDNF treatments also reduced body weights in the rats of Wistar-BDNF and GK-BDNF groups (Figure 5B). After termination of treatment, the body weight of the BDNF-treated groups rebounded to the level close to that before BDNF treatment (Figure 5B). Although the blood glucose level in GK-BDNF level did not change during BDNF treatment, after termination of treatment it became lower than that in GK-Vehicle group, demonstrating the late-onset effect of BDNF on glucose control (Figure 6). To investigate the mechanism how BDNF lowered the casual glucose level in GK-BDNF group, we performed IpGTT and measured glucose and insulin levels during IpGTT (Figure 7). Blood glucose levels during IpGTT in GK-Vehicle and GK-BDNF groups were markedly higher than that in Wistar-BDNF groups at Day 0 (before treatment), Day 7 (immediately after termination of treatment), and Day 11 (after termination of treatment) (Figure 7A). Blood glucose level during IpGTT at Day 7 tended to increase, rather than decrease, in GK-BDNF group. The higher glucose level at Day 7 in GK-BDNF group is probably due to the lowered insulin level at Day 7 (Figure 7B). These results taken together suggest that BDNF treatments lowered casual blood glucose level by changing insulin sensitivity but not by improving insulin secretion in GK rats. To examine whether exogenous BDNF treatment changes plasma NEFA level in GK rats, we measured plasma NEFA level in fed and 12h-fasted conditions. In GK-Vehicle group, plasma NEFA level was higher in fasted condition at all time points measured (Figure 8). In GK-BDNF and Wistar-BNDF groups, plasma NEFA level was significantly increased both in fed and fasted conditions at Day 6(fed)/7(fasted) and returned to pretreatment level at Day 10 (fed)/11(fasted) (Figure 8), indicating the possibility that BDNF not only works as a feeding suppressant but also increases lipolysis in GK rats.

### Comparison of plasma leptin levels between GK-BDNF and GK-Vehicle pair-fed groups

**Figure 9:**
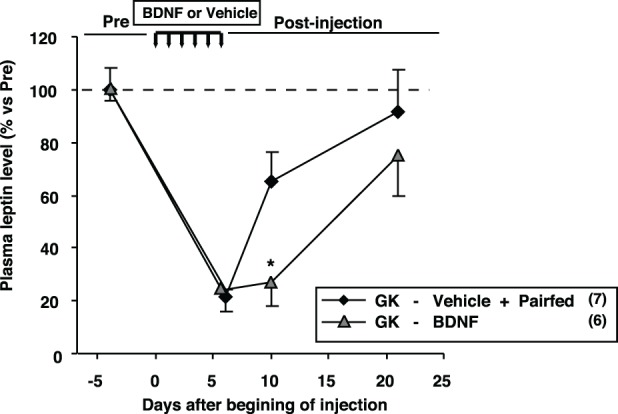
**Comparison of plasma leptin levels between GK-BDNF and GK-Vehicle pair-fed groups.** In both groups, plasma leptin levels at Day 6 were decreased after BDNF and pair-fed treatments. At Day 10, plasma leptin level in GK-BDNF group remained low while that in GK-Vehicle pair-fed group was significantly elevated (^*^p < 0.05, Student's *t*-test).

We examined plasma leptin levels in GK-BDNF and GK-Vehicle pair-fed groups (Figure 9). In GK-Vehicle pair-fed group, GK rats were treated with saline and the amount of food supplied was controlled to the same level as consumed by GK-BDNF group. In GK-BDNF group, at Day 6 and 10 plasma leptin levels were markedly reduced (Figure 9). In GK-Vehicle pair-fed group, plasma leptin level was lowered to the same level as in GK-BDNF group at Day 6. However, at Day 10 it increased to a level significantly higher than that in GK-BDNF group, indicating that exogenous BDNF counteracted visceral adiposity and hyperleptinemia via two modes of action: in acute phase via anorexigenic action and in late long-lasting phase via food intake-independent mechanisms.

## Discussion

In this study, we found that middle-aged GK rats show abdominal fat accumulation and hyperleptinemia, reduction in BDNF mRNA expression and protein levels specifically in VMH, and reduction in GLUT2 mRNA in VMH. Pharmacological blockade of glucose utilization *in vivo* and *in vitro* reduced BDNF expression in VMH, suggesting that glucose availability positively regulates BDNF expression in VMH. In rescue experiment, BDNF supplementation for 6 days ameliorated hyperleptinemia and higher adiposity in a long-lasting manner. These results reveal that reduction of BDNF expression due to decreased GLUT2 expression and glucose utilization in VMH is linked to visceral fat accumulation and hyperleptimemia in GK rats.

### Fat accumulation and glucose intolerance in middle-aged GK rats

It has been reported that glucose intolerance in human type 2 diabetes progresses with age (Gong and Muzumdar, 2012). A major cause for this progression is insulin resistance due to genetic and/or environmental factors. Especially, visceral fat accumulation is of particular concern as a causal factor to induce insulin resistance (Pouliot et al., 1992). Our breeding colony of GK rats displayed these characteristic features common for the type 2 diabetes. First, hyperglycemia and glucose intolerance progressed markedly at 24 weeks of age compared to 14 weeks (Figure A1). Second, the visceral adiposity in mesenteric and perirenal fat pads in GK rats was greater at 26 weeks of age than at 11 weeks. Third, progression of hyperglycemia paralleled with progression of adiposity. These findings suggested that higher visceral adiposity is related to the progression of glucose intolerance and that GK rat is a suitable animal model to study interaction of glucose intolerance and visceral adiposity in type 2 diabetes.

### Reduction of BDNF expression in VMH of GK rats at middle age

BDNF in the brain plays a critical role in regulating feeding and metabolism. The BDNF neurons expressed in VMH and PVN have been reported to contribute to the regulation of feeding and metabolism (Noble et al., 2011). We found that the GK rats aged 26 weeks display reduction of the BDNF mRNA and protein in the VMH in parallel with progression of higher adiposity. Regarding regulation of BDNF, it has been reported that BDNF mRNA expression is modulated by the melanocortine receptor 4 (MC4R) and downstream pathway (Xu et al., 2003; Nicholson et al., 2007). Therefore, we examined a possible alteration of the α-MSH-MC4R system as a cause of BDNF reduction in GK rats. However, neither the number of α-MSH-immunoreactive neurons nor the MC4R mRNA levels in the ARC and VMH were altered in GK rats at 26 weeks of age. We also examined whether insulin and/or leptin influence BDNF expression in VMH. We could not find convincing evidence that insulin and leptin regulate BDNF mRNA level in GK and Wistar rats.

We found that the GLUT2 mRNA level in VMH was significantly reduced in GK rats. Previous studies in GK rats reported reductions of GLUT1 in the retina (Fernandes et al., 2004), GLUT2 in the pancreatic islets (Ohneda et al., 1993) and GLUT4 in the heart (Desrois et al., 2004). Glucose uptake in the skeletal muscle was also reported to be impaired in GK rats (Krook et al., 1997). As a causal factor of GLUT reduction, it has been implicated that over-activation of hexosamine pathway by hyperglycemia decreases the expression level of GLUT2 in the pancreatic β-cells, eventually leading to the inhibition of glucose-stimulated insulin secretion and induction of apoptosis in β-cells (Yoshikawa et al., 2002). The down-regulation of GLUT2 is expected to suppress the glucose uptake and utilization in a specific subset of neurons presumably including BDNF-expressing neurons, which could suppress BDNF mRNA in the VMH of GK rats. In support of this speculation, icv injection of 2-DG in Wistar rats, a procedure used to impair the glucose utilization and mimics the impairment in GK rats, significantly decreased BDNF mRNA level in the VMH. Furthermore, the bath application of 2-DG to the cells isolated from the medial basal hypothalamus including VMH, significantly decreased BDNF mRNA level. Our *in vitro* results demonstrate that BDNF expression in VMH is directly regulated by glucose availability within BDNF neurons. Oomura et al. originally found subpopulations of neurons in the VMH that respond to hyperglycemic or hypoglycemic condition (Oomura et al., 1969; Mizuno and Oomura, 1984; Nakano et al., 1986). Experimental cytosolic Ca^2+^ imaging combined with single-cell RT-PCR showed that up to 60% of glucose-sensing neurons in the VMH express glucokinase mRNA (Kang et al., 2004). It could be speculated that BDNF neurons in VMH have machinery to sense extracellular glucose level and change BDNF mRNA expression. GLUT2 and glucokinase are the candidate machineries. It has been reported that GLUT2 is expressed in a specific subset of VMH neurons in the brain

(Arluison et al., 2004) and certain population of GLUT2-expressing neurons in VMH have a property to respond to change of extracellular glucose concentration (Kang et al., 2004). Considering that both GLUT2-expressing and BDNF-expressing neurons are able to respond to glucose, the two populations could be overlapped. This hypothesis should be examined by either double immunostaining or *in situ* hybridization of BDNF and GLUT2 in future study. Taken together, our finding demonstrates the possibility that the BDNF mRNA expression in the VMH is controlled by glucose metabolism in which glucokinase and GLUT2 might play a role.

In middle-aged GK rats with reduced level of BDNF in VMH, hyperphagia was not found as shown in previous study (Maekawa et al., 2006a). On the other hand, it has been reported that the reduced level of BDNF in medial basal hypothalamus by conditional BDNF gene knockout causes hyperphagia (Unger et al., 2007). What causes the difference between these phenotypes? It has been reported that the BDNF neurons in VMH regulate various physiological functions such as feeding, energy expenditure, and blood glucose control (Noble et al., 2011). It could be speculated that each function is assigned to a specific subgroup of BDNF-expressing neurons in VMH. Since the BDNF-expressing neurons at the level up to 30% of that in Wistar rats persisted in middle-aged GK rats, such subgroup of BDNF neurons might play a role in preventing hyperphagia whereas the subgroup might take no part in the regulation of lipolysis. This possibility should be validated in future study.

### Central BDNF supplementation ameliorated higher adiposity in GK rats

It has been reported that lesioning the VMH induces not only hyperphagia but also a marked visceral fat accumulation in Wistar (Bray and Nishizawa, 1978) and GK rats (Yoshida et al., 1996), and that the loss of neurons in the VMH induces dysfunction of lipolysis. The neurons in VMH are known to project to various brain areas. In our previous report using Wistar rats (Maekawa et al., 2006b), only 19% of BDNF neurons of VMH project to midbrain central gray, a region known to be innervated by VMH neurons. PVN is one of possible candidates for primary synaptic transmission of VMH BDNF neurons. Our previous report suggested that TrkB receptor, a neurotrophin receptor specific for BDNF, is expressed in CRH neurons of PVN and icv BDNF injection increases the CRH mRNA in PVN (Toriya et al., 2010). Although there is no direct evidence that BDNF neurons of VMH are connected to CRH neurons in PVN, these results obtained by physiological experiments indirectly support the possibility that the VMH BDNF neurons project to CRH neurons in PVN. It has been demonstrated that CRH neuron in PVN regulates both sympathetic tone and hypothalamic-pituitary-adrenal axis and that CRH neurons positively control lypolysis (Yada et al., 2012). Our previous report demonstrated that lypolytic effect of BDNF was countracted by simultaneous treatment with CRH receptor antagonist, suggesting that the lypolytic effect of BDNF is mediated by CRH release from CRH neurons in PVN (Toriya et al., 2010). Thus, our present and previous results taken together suggest a possibility that the reduction of BDNF in VMH impairs lipolysis via reducing CRH release in GK rats. Other than CRH release, TrkB and CRH receptor in hypothalamus including PVN might be changed concomitantly with the change in CRH release in GK rats. Such possibilities should also be taken into account when elucidating the etiology of visceral fat accumulation in GK rats.

BDNF has been reported to regulate the binding of cAMP response-element binding protein (CREB) and coactivator proteins to CRH promoter and thereby positively control transcription of CRH (Jeanneteau et al., 2012). Activation of phospholipase Cγ might mediate the BDNF-TrkB signaling by triggering Ca^2+^ increase, activating adenylate cyclase and subsequently increasing cAMP concentration (Ji et al., 2005). On the other hand, BNDF works not only as a neurotransmitter but as a regulator of synaptic structural plasticity (Yoshii and Constantine-Paton, 2010). The regulation includes spine formation by altered localization of postsynaptic proteins such as PSD-95 (Yoshii et al., 2003). Regulation of synaptic plasticity by BDNF, a process thought to be requisite for learning both at juvenile and adult ages (Poo, 2001; Lu et al., 2008; Suzuki et al., 2012), might also be related to the control of feeding and energy metabolism.

### Future perspective

The molecular mechanisms connecting lower glucose metabolism to BDNF reduction remained unclear in this study. One of key proteins that link glucose metabolism to BDNF expression is SIRT1, a metabolic sensor by working as a NAD^+^-dependent deacetylase. It has been reported that SIRT1 controls BDNF expression by deacetylating MeCP2 (Zocchi and Sassone-Corsi, 2012), by enhancing CREB-TORC1 transcriptional activity (Jeong et al., 2011), and by suppressing expression of specific microRNA which binds to BDNF mRNA (Gao et al., 2010). Another possible protein to link glucose metabolism to BDNF expression is neuron-restrictive silencer factor (NRSF). The NRSF, a transcriptional repressor, has been reported to recruit the NADH-binding co-repressor CtBP to BDNF promoter. Notably, it has been also reported that glycolysis inhibition by 2-DG injection reduces BDNF transcription by NRSF-CtBP-dependent change of histone modification (Garriga-Canut et al., 2006). Involvements of such proteins to BDNF reduction should be examined in future study.

In this study, we found that BDNF treatments reduce fat mass and lower casual blood glucose level by changing insulin sensitivity. However, BDNF could not restore the insulin secretion in GK rats. Therefore, to improve the glycemic control in type-2 diabetes more effectively, the simultaneous treatment with BDNF and the drug having a function to alleviate impaired insulin secretion might be required. Therapeutic potential of the combination of BDNF with the drug enhancing insulin secretion against diabetes should be investigated in future study.

## Conclusion

In this study, we present the following scenario to explain how the higher visceral adiposity continues in GK rats until middle-aged adult. (1) The hyperglycemia and/or other diabetes-associated factors suppress the GLUT2 expression to lower the glucose availability in VMH. (2) The reduction of glucose availability leads to impair BDNF expression in VMH. (3) The reduced BDNF level attenuates lypolysis and accelerates glucose intolerance. Further studies are required to validate whether this scenario in GK rats is more generally applicable to other diabetes and/or obesity models.

### Conflict of interest statement

The authors declare that the research was conducted in the absence of any commercial or financial relationships that could be construed as a potential conflict of interest.

## Appendix

**Table A1:** **Primer sequences used for real-time RT-PCR**.

**mRNA (Abbreviation)**	**Genbank accession number (amplicon)**	**Primers**	**Probe (5′-FAM, 3′-TAMRA)**
Brain-derived neurotrophic factor (BDNF)	NM012513 (504-571)	Fwd: CCATAAGGACGCGGACTTGTAC	CTTCCCGGGTGATGCTCAGCAGT
		Rvs: GAGGAGGCTCCAAAGGCACTT	
Melanocortin-4 receptor (MC4R)	NM013099 (731-819)	Fwd: TGGCGAGGCTTCACATTAAGA	CACGGGTACCATCCGACAGGGTG
		Rvs: CAAGGTAATTGCGCCCTTCA	
Glucose transporter-2 (GLUT2)	NM012879 (818-898)	Fwd: GTCCAGAAAGCCCCAGATACC	TTGCCCTGACTTCCTCTTCCAAATTTAGGT
		Rvs: TGCCCCTTAGTCTTTTCAAGCT	
Glucose transporter-4 (GLUT4)	NM012751 (818-907)	Fwd: CCCCCGATACCTCTACATCATC	CTGCCCGAAAGAGTCTAAAGCGCCT
		Rvs: GCATCAGACACATCAGCCCAG	
Glucose transporter-8 (GLUT8)	NM053494 (1049-1235)	Fwd: TCATGGACAGAGCAGGGCG	Not used
		Rvs: GCCAGCCAGGCCAGCCCCA	
Glucokinase	NM012565 (1330-1414)	Fwd: CAAGCTGCACCCGAGCTT	TCAGCCTGCGCACACTGGCG
		Rvs: TGATTCGATGAAGGTGATTTCG	
